# Supplementary material for: Emotional Eating in Relation to Worries and Psychological Distress Amid the COVID-19 Pandemic: A Population-Based Survey on Adults in Norway
Source: Int J Environ Res Public Health. 2020 Dec 27;18(1):130. doi: 10.3390/ijerph18010130 (PMC7795972; doi:10.3390/ijerph18010130)
Supplement: Supplementary file 1 [file ijerph-18-00130-s001.zip › ijerph-1039879-Document S1.pdf]

Supplementary Material—Document S1

Questionnaire items

Item focusing on emotional eating

Q: Have you, during the past week, engaged in comfort eating or eating extra in response to feeling unhappy or unsatisfied?

A: 1 (never) – 2 – 3 – 4 – 5 – 6 – 7 (every day)

Items focusing on health-related worries

Q: I have become scared and anxious (worried) that the infection will affect myself

A: Not true – somewhat true – completely true

Q: I have become scared and anxious (worried) that the infection will affect one of my closest ones

A: Not true – somewhat true – completely true

Q: I have become scared and anxious (worried) that the infection will affect elderly members of my family

A: Not true – somewhat true – completely true

Items focusing on worries regarding personal economy:

Q: I fear (am worried) that the outbreak will cause me to be laid-off temporarily or lose my job

A: Not true – somewhat true – completely true

Q: I fear (am worried) that the outbreak will lead to a poorer personal economy

A: Not true – somewhat true – completely true

Items focusing on consumption of sugar-containing food items and beverages

Q: How often, during the past month, did you eat or drink the following

Q1: Sugar-containing soda or soft drink

A1: Rare/never – 1-3 times/month – 1-3 times/week – 4-6 times/week – daily

Q2: Candy, cakes, biscuits or sweet desserts

A2: Rare/never – 1-3 times/month – 1-3 times/week – 4-6 times/week – daily
