# Supplementary material for: Emotional Eating in Relation to Worries and Psychological Distress Amid the COVID-19 Pandemic: A Population-Based Survey on Adults in Norway
Source: Int J Environ Res Public Health. 2020 Dec 27;18(1):130. doi: 10.3390/ijerph18010130 (PMC7795972; doi:10.3390/ijerph18010130)
Supplement: Supplementary file 1 [file ijerph-18-00130-s001.zip › ijerph-1039879-Table S1.pdf]

**Table S1**– Multinomial logistic regression of emotional eating by predictors.

| | Relative risk ratios | | | | | |
|---|---|---|---|---|---|---|
| | 2 | 3 | 4 | 5 | 6 | 7 |
| **Gender** | | | | | | |
| Male | Ref. | Ref. | Ref. | Ref. | Ref. | Ref. |
| Female | 1,7** | 2,1** | 2,3** | 2,1** | 2,9** | 2,6** |
| **Age** | | | | | | |
| 18-29 | Ref. | Ref. | Ref. | Ref. | Ref. | Ref. |
| 30-39 | 1,1 | 1,0 | 1,2* | 1,3** | 1,4* | 2,0** |
| 40-49 | 1,0 | 1,0 | 1,1 | 1,0 | 1,1 | 1,7** |
| 50-59 | 1,0 | 0,9* | 0,9 | 0,7** | 0,8 | 1,4* |
| 60-69 | 0,9* | 0,8* | 0,7** | 0,5** | 0,4** | 0,8 |
| 70+ | 0,7** | 0,5** | 0,5** | 0,3** | 0,2** | 0,4** |
| **Education level** | | | | | | |
| Primary school | Ref. | Ref. | Ref. | Ref. | Ref. | Ref. |
| High or trade school | 1,0 | 1,1 | 1,0 | 1,1 | 1,2 | 1,0 |
| ≤ 3 years of higher education | 1,1 | 1,1 | 1,1 | 1,2 | 1,3 | 0,8 |
| ≥ years of higher education | 1,2* | 1,1 | 1,0 | 1,2 | 1,3 | 0,7* |
| **Quarantined** | | | | | | |
| No | Ref. | Ref. | Ref. | Ref. | Ref. | Ref. |
| Yes | 1,1* | 1,3** | 1,2* | 1,3** | 1,2* | 1,4* |
| **Temporarily laid-off** | | | | | | |
| No | Ref. | Ref. | Ref. | Ref. | Ref. | Ref. |
| Yes | 1,2* | 1,0 | 0,9 | 1,1 | 1,0 | 0,9 |
| **Health-related worrie** | | | | | | |
| None | Ref. | Ref. | Ref. | Ref. | Ref. | Ref. |
| Some | 1,2* | 1,5** | 1,4** | 1,4** | 1,3* | 1,2* |
| Substantial | 1,1 | 1,5** | 1,4** | 1,5** | 1,4* | 1,6** |
| **Economy-related worries** | | | | | | |
| None | Ref. | Ref. | Ref. | Ref. | Ref. | Ref. |
| Some | 1,1 | 1,4** | 1,5** | 1,3** | 1,6** | 1,5** |
| Substantial | 1,0 | 1,4** | 1,8** | 1,7** | 2,3** | 3,0** |
| **Psychological distress** | | | | | | |
| SCL10 ≤ 1,85 | Ref. | Ref. | Ref. | Ref. | Ref. | Ref. |
| SCL10 ≥ 1,85 | 1,9** | 3,5** | 4,7** | 6,7** | 9,9** | 12,5** |

*significant at $p < 0.05$
**significant at $p < 0.001$
